# Emotion Recognition Using Smart Watch Sensor Data: Mixed-Design Study

**DOI:** 10.2196/10153

**Published:** 2018-08-08

**Authors:** Juan Carlos Quiroz, Elena Geangu, Min Hooi Yong

**Affiliations:** ^1^ Department of Computer Science and Engineering University of Nevada Reno, NV United States; ^2^ Australian Institute of Health Innovation Macquarie University NSW Australia; ^3^ Department of Psychology University of York York United Kingdom; ^4^ Department of Psychology Sunway University Bandar Sunway Malaysia

**Keywords:** emotion recognition, accelerometer, supervised learning, psychology

## Abstract

**Background:**

Research in psychology has shown that the way a person walks reflects that person’s current mood (or emotional state). Recent studies have used mobile phones to detect emotional states from movement data.

**Objective:**

The objective of our study was to investigate the use of movement sensor data from a smart watch to infer an individual’s emotional state. We present our findings of a user study with 50 participants.

**Methods:**

The experimental design is a mixed-design study: within-subjects (emotions: happy, sad, and neutral) and between-subjects (stimulus type: audiovisual “movie clips” and audio “music clips”). Each participant experienced both emotions in a single stimulus type. All participants walked 250 m while wearing a smart watch on one wrist and a heart rate monitor strap on the chest. They also had to answer a short questionnaire (20 items; Positive Affect and Negative Affect Schedule, PANAS) before and after experiencing each emotion. The data obtained from the heart rate monitor served as supplementary information to our data. We performed time series analysis on data from the smart watch and a *t* test on questionnaire items to measure the change in emotional state. Heart rate data was analyzed using one-way analysis of variance. We extracted features from the time series using sliding windows and used features to train and validate classifiers that determined an individual’s emotion.

**Results:**

Overall, 50 young adults participated in our study; of them, 49 were included for the affective PANAS questionnaire and 44 for the feature extraction and building of personal models. Participants reported feeling less negative affect after watching sad videos or after listening to sad music, *P*<.006. For the task of emotion recognition using classifiers, our results showed that personal models outperformed personal baselines and achieved median accuracies higher than 78% for all conditions of the design study for binary classification of happiness versus sadness.

**Conclusions:**

Our findings show that we are able to detect changes in the emotional state as well as in behavioral responses with data obtained from the smartwatch. Together with high accuracies achieved across all users for classification of happy versus sad emotional states, this is further evidence for the hypothesis that movement sensor data can be used for emotion recognition.

## Introduction

Our emotional state is often expressed in a variety of means, such as face, voice, body posture, and walking gait [1,2]. Many studies are conducted in strict laboratory settings, which may impede the overall ecological validity of the findings. Having a strong ecological validity is important because emotional expression or display in any modality is not entirely dependent on conscious action or function. Instead, emotional expressions are essentially a response to a particular affective stimulus or experience, and this response might be reduced in a laboratory as a result of social desirability.

Speech, video, and physiological data have been analyzed to determine the emotional state of a person [3,4], but these analyses usually rely on recordings obtained in laboratory environments with limited ecological validity. To formulate theoretical models of emotions and affective health that take into account the richness of everyday life, we need to measure affective states unobtrusively. Mobile phones include sensors, such as accelerometers, that have the potential to be sensitive to changes in people’s affective states and thus could provide rich and accessible information in this respect; for example, we know that the way we walk reflects whether we feel happy or sad [2]. This paper analyzes movement sensor data recorded via a smart watch in relation to changes in emotions.

Prior work on emotion detection from mobile phone data includes analysis of typing behavior on a mobile phone [5,6] and mobile phone usage [7,8]. The EmotionSense system performed emotion detection directly on mobile phones via analysis of speech with additional sensors collecting information about the user and the environment [9]. However, there are some indications that movement sensor data collected by mobile phones could be a viable solution for inferring emotion, as opposed to inferring movement or physical activities. Cui et al attempted to record participants’ movements with mobile phones strapped to their ankles and wrists, thus impairing ecological validity [10]. Happiness and anger were elicited with video stimuli, and emotional state classifiers were trained with accelerometer data [10]. Zhang et al also focused on happiness and anger, but they recorded movement data with smart bracelets [11]. Accuracies in detecting these emotions ranged from 60% to 91.3% across all subjects using 10-fold cross-validation [11].

These cases have motivated further research on tracking and analysis of sensor data from mobile phones and wearables with the goal of monitoring and intervening for patients suffering from mental illnesses or substance abuse [12,13]. Further validation is needed for the hypothesis that movement sensor data can be used to recognize emotional states. Movement data are of particular interest because accelerometers and gyroscopes are standard sensors in mobile phones, wearables, and fitness trackers. Movement data collection is unobtrusive, and it requires no user input [14], which gives us reliable data in the real world without the possibility of having social desirability responses.

**Figure 1:**
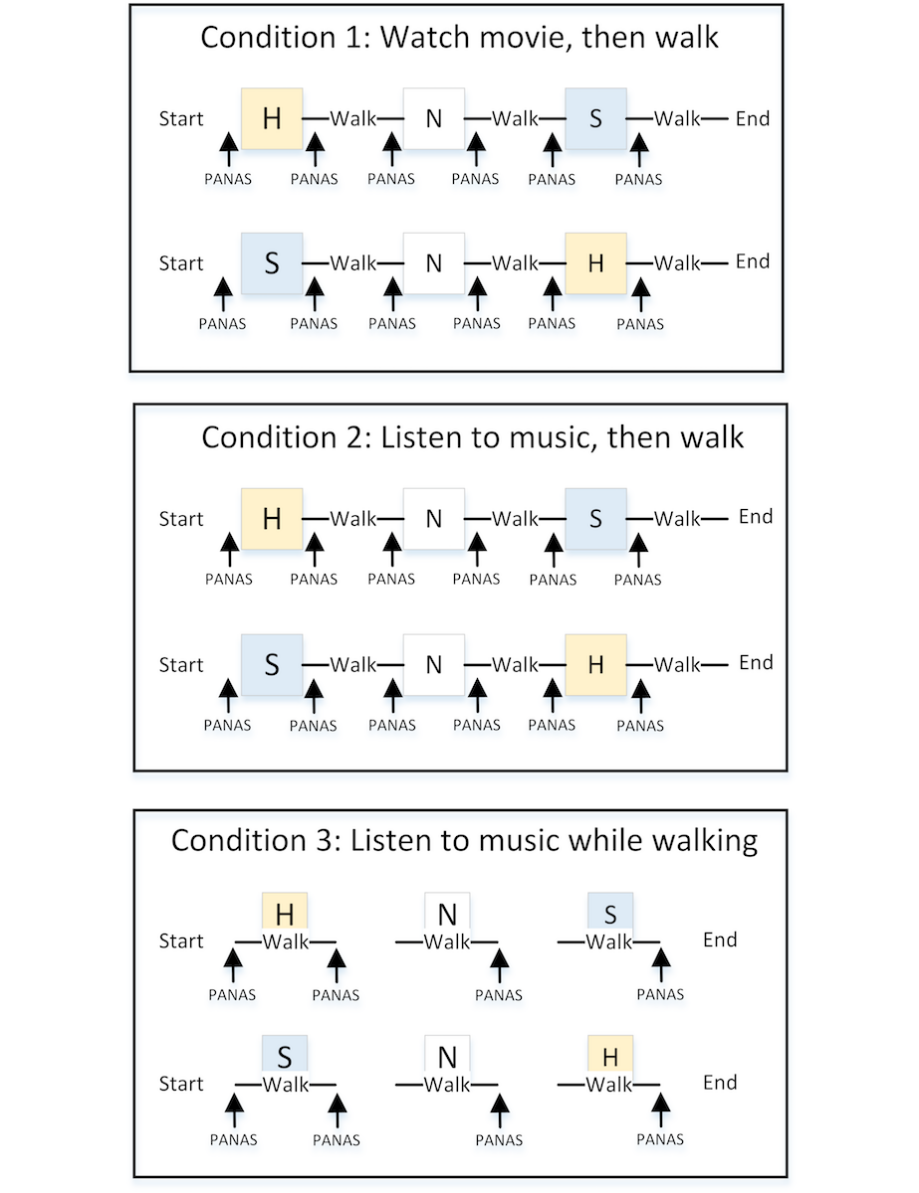
Mixed-design study with three conditions. The three conditions were used to determine the stimulus that would better induce the target emotional states on participants. PANAS: Positive Affect and Negative Affect Schedule.

Toward the end, we make the following contributions. First, we conducted a mixed-design study, as seen in Figure 1, with 50 participants to test two types of stimuli, audiovisual and audio, for eliciting emotional responses from participants. Participants wore a smart watch on the wrist and a heart rate strap on the chest. The heart rate strap was included to supplement data collected from Positive Affect and Negative Affect Schedule (PANAS) scores [15]. After or while watching emotional stimuli, participants walked, and the process was repeated three times, for each of the following emotions: happy, neutral, and sad. We extracted features from sensor data and built classifiers (personal models) that recognized the emotional state of the user. Our results show that personal models outperformed personal baselines and achieved median accuracies higher than 78% for all conditions of the design study for binary classification of happiness versus sadness. This paper is an extended version of preliminary findings published [16].

## Methods

### Participants

In total, 50 young adults participated in this study (43 females; mean age 23.18 [SD 4.87] years). All participants were recruited in a university campus (North-West UK) via announcements on notice boards and by word of mouth. Each participant was given £7 for participation. None of the participants reported any vision or hearing difficulties and could walk unassisted.

### Ethics Approval

We obtained ethical approval from Sunway University Ethics Board (SUREC 2016/05) and had it validated by Lancaster University to conduct both validation and the actual main study experiment.

### Materials

The study included the following two types of stimuli: a) audiovisual and b) audio.

#### Audiovisual

Audiovisual clips were selected from commercial movies with the potential of being perceived as having emotional meaning (ie, sadness and happiness) and able to elicit emotional responses. Commercial movies were selected from Gross and Levenson [17], Bartollini [18], Schaefer et al [19], and from 5 young adults (4 females; mean age 21.50 years). Another 5 participants (3 females; mean age 22.80 [SD 1.30] years) were asked to identify each of these clips in terms of the emotion they felt while watching, and the intensity of the emotion they felt using a 0-to-10 Likert scale (0: hardly; 10: very much likely). They were also asked if they had watched that movie before. On average, only one participant had seen that movie before. Participants reported that they felt the emotion intended for all clips (100% accuracy) and the intensity experienced ranged between 5.0 to 6.5 for happy and sad clips, respectively. Table 1 includes the movie clips used in our study.

#### Audio

For audio stimuli, pieces of classical music known to elicit happy, sad, and emotionally neutral states were chosen [20]. Table 2 includes selected clips.

### Procedure

All participants were presented with happy, sad, and neutral stimuli. A third of the participants (n=18) were presented with audiovisual stimuli (ie, videos), whereas the other participants (n=32) were presented with audio stimuli (ie, classical music). Half the participants (n=16), who were assigned to audio stimuli, listened to them prior to walking, whereas the other half (n=16) listened to stimuli while they were walking. Eighteen participants (n=18) who were assigned to watch emotional videos watched them prior to walking. Assignment to each condition was random. To counter possible order effects, half the participants were presented with sad stimuli first, whereas the other half were presented with happy stimuli first. Each participant was tested individually, and the task took approximately 20 minutes to complete. All data was collected between 17:00 and 19:00 h to account for peak foot traffic.

**Table 1:** Movies used to induce happy and sad emotions.

Emotion and movie		Scene
**Happy**		
	*10 Things I Hate About You* (1999)	Patrick serenades Katarina in stadium
	*When Harry Met Sally* (1989)	Discussion of orgasms in cafe
	*There’s Something about Mary* (1998)	Mary hair gel scene
	*Monty Python* (1975)	Black Knight fights King Arthur
	*Modern Times* (1936)	Factory worker in assembly line
	*Love Actually* (2003)	Arrival halls scene in Heathrow airport
	*Wall-E* (2008)	EVA kisses Wall-E
	*Benny & Joon* (1993)	Sam roll dance in diner
**Sad**		
	*Interstellar* (2014)	Cooper watches video messages sent by his children
	*Click* (2006)	Michael rewinds his past to recall not saying goodbye to his father
	*Hachi* (2009)	Hachiko waits at the train station
	*Shawshank Redemption* (1994)	Death of Brooks
	*Saving Private Ryan* (1998)	Mother is informed of the deaths of all of Private Ryan’s brothers
	*Marley & Me* (2008)	Marley is euthanized in the veterinarian clinic
	*The Champ* (1979)	Boy cries at father’s death
	*My Girl* (1991)	Thomas’s funeral

**Table 2:** Musical pieces used to induce happy and sad emotions and neutral ones.

Emotion and piece		Composer	
**Happy**			
	*Carmen*: “Chanson du Toreador”		Bizet
	“Allegro”—*A Little Night Music*		Mozart
	“Rondo Allegro”—*A Little Night Music*		Mozart
	“Blue Danube”		Strauss
	“Radetzky March”		Strauss
**Sad**			
	*Adagio in Sol Minor*		Albinoni
	“Kol Nidrei”		Bruch
	“Solveig’s song”—*Peer Gynt*		Grieg
	*Concierto de Aranjuez*		Rodrigo
	*Suite for violin & orchestra in A minor*		Sinding
**Neutral**			
	“L’oiseau prophete”		Schumann
	“Au Clair de lune”		Beethoven
	“Clair de lune”		Debussy
	*Symphony no. 2 in C minor*		Mahler
	*La Traviata*		Verdi
	*Pictures at an Exhibition*		Mussorgsky
	“Water Music Suite: 5. Passepied”		Handel
	“Violin Romance no. 2 in F major”		Beethoven
	“Water Music”—minuet		Handel
	*The Planets* —“Venus”		Holst

The three conditions of the mixed-design study are presented in Figure 1 and are as follows: Condition 1—watching the movie clip prior to walking; Condition 2—listening to the music prior to walking; and Condition 3—listening to the music while walking.

Each participant was first greeted by the experimenter at one end of the corridor and was helped to put on various items. First, the participant had the heart rate sensor (Polar H7) strapped snugly around the chest. The corresponding watch (Polar M400) was strapped onto the experimenter’s wrist. The watch was set to the “other indoor” sport profile. Second, the participant strapped a smart watch (Samsung Gear 2) on the left wrist. Participants wore sensors for the entire duration of the experiment. The smart watch included a triaxial accelerometer and a triaxial gyroscope. The sampling rate of the smart watch is advertised as 25 Hz, but our results show that the actual sampling rate on average was 23.8 Hz. For the smart watch, we developed a Tizen app that recorded accelerometer and gyroscope sensor data.

Participants rated their current mood state using PANAS [21] on a 7-inch tablet. PANAS contains 10 adjectives for positive (eg, joy) and 10 adjectives for negative feelings (eg, anxiety). Scores can range from 10 to 50 with higher scores representing higher levels of affect. The heart rate sensor was used in the study to supplement data collected from PANAS scores [15].

For Conditions 1 and 2, in which the stimulus presentation occurred before walking, participants wore a pair of headphones to listen or watch the assigned stimuli (eg, sad music or happy movie) while at the start of a walking route. At the end of the stimulus, the participant walked to the end of the route and back to the starting point. Participants were reminded not to make any stops in between. The route was represented by a 250 m S-shaped corridor located on the ground floor of a university building. The experimenter discreetly followed the participant at a 125 m distance to observe the behavior and to ensure that heart rate monitoring was captured by the watch. Upon return, participants rated their mood using the same PANAS scales. Because of the initial mood induction, we always had a neutral condition between happy and sad conditions to allow return to the baseline calm state. For all participants, the neutral stimulus was classical music for the audio type or a movie with classical music playing in the background and depicting an everyday scene. The same procedure above, rating their initial mood using PANAS, watching or listening to a stimulus, walking along the corridor and back, and rating their mood, was applied to the neutral and second emotion.

In Condition 3, which included listening while walking, the procedure was similar as above, except that the participant was listening to the assigned music while walking, and participants reported PANAS scores after walking.

### Feature Extraction

During the experiment, the experimenter recorded the time each participant started and stopped walking. These times were used to identify sensor data that corresponded to the actual walking time. We discarded sensor data when participants were briefed and when participants watched or listened to the stimulus prior to walking.

The walking times were labeled according to the corresponding emotional stimulus presented before walking; for example, if the participant viewed a movie clip known to induce happiness, all of the features extracted from the subsequent walking data were labeled as happy. These labels were used to train classifiers for the recognition of happiness versus sadness. We present classifier results for the two-class problem of detecting happy versus sad emotions and for the three-class problem of detecting happy versus sad versus neutral emotions.

We first filtered raw accelerometer data with a mean filter (window=3). Features were extracted from sliding windows with a size of one second (24 samples) with 50% overlap. Our feature extraction approach is similar to that used for activity recognition from mobile phone accelerometer data [22,23], that is, each window is treated as an independent sample (feature vector). We address neighborhood bias when building models from accelerometer sliding windows in the results section [24].

For each window of the triaxial accelerometer and triaxial gyroscope data, we extracted 17 features [23]: (1) mean, (2) SD, (3) maximum, (4) minimum, (5) energy, (6) kurtosis, (7) skewness, (8) root mean square, (9) root sum square, (10) sum, (11) sum of absolute values, (12) mean of absolute values, (13) range, (14) median, (15) upper quartile, (16) lower quartile, and (17) median absolute deviation. These 17 features were extracted from each of the 3 axes of the accelerometer data and each of the 3 axes of the gyroscope data, resulting in 102 features. We also calculated the angle between the signal mean (within a window) and the x-axis, y-axis, and z-axis (3 features); SD of signal magnitude (one feature); and the heart rate (one feature) for a total of 107 features for the feature vector of a window. Unless stated otherwise, we used all 107 features for classification. However, we do explore classification performance based on features corresponding to certain sensors: accelerometer, gyroscope, and heart rate; accelerometer and heart rate; and accelerometer.

We divided data by condition and built personal models with features extracted from each window [25]. In personal models, training and testing data come from a single user. In our case, we built 44 personal models (data from 6 participants were discarded because of missing data and other recording errors) with each model evaluated using stratified 10-fold cross-validation that was repeated 10 times. For each participant, we obtained a mean of 403.29 (55.62) samples labeled as happy, 403.67 (51.46) samples labeled as sad, and 402.93 (50.24) samples labeled as neutral. Of the 44 personal models built, 16 were from Condition 1 (watch movie and then walk), 14 were from Condition 2 (listen to music and then walk), and 14 were from Condition 3 (listen to music and then walk).

We compared random forest models, with 100 estimators and logistic regression, with L2 regularization and a baseline classifier that picked the majority class as the prediction. The python scikit-learn library was used for training and testing these classifiers. Because the number of samples labeled as happy versus sad for each participant was approximately the same, the baseline classifier predicted each window as happy versus sad with about a 50% probability (ie, all samples for user *i* were classified as happy, resulting in about 50% accuracy). For binary classification of happy versus sad, we use the accuracy, the F1 score, and the area under the receiver operating characteristic curve (ROC AUC) to assess classification performance. For multiclass classification of happy versus sad versus neutral, we use the accuracy and the F1 score.

## Results

### Ecological Validity Checks

When asked about their experience in using a smart gadget, most participants were familiar and comfortable with the smart watch but not with the Polar heart rate monitor. They did not notice anything unusual about the study that might have influenced their walking gait and behavioral response.

### Behavioral Response to Stimuli (Positive Affect and Negative Affect Schedule Outcomes)

We analyzed PANAS responses for all conditions on the happy versus sad stimuli. One participant’s data was excluded for being incomplete, thus leaving 49 for analyses (15 for Condition 1, 18 for Condition 2, and 16 for Condition 3). We first reviewed normality and found that data was normally distributed for Conditions 1 and 2 but not for Condition 3 (visual histograms were skewed and Shapiro-Wilk *P*<.01). See Multimedia Appendix 1 for PANAS scores for each emotion.

#### Condition 1: Watch Movie and Then Walk

Participants reported a reduced negative affect after watching a sad movie clip (mean 14.94 [SD 6.79]) compared with that before watching it (mean 19.00 [SD 7.20], *t*_16_=3.16, *P*=.006). There was no significant difference for the positive affect for the sad movie (*t*_16_=.08, *P*=.94) and for both affects with respect to the other two emotions (happiness and neutral), all *P* values were >.10.

#### Condition 2: Listen to Music and Then Walk

For sad music, participants reported an increased positive effect after the walk (mean 24.00 [SD 5.33]) compared with that before watching it (mean 20.31 [SD 5.79], *t*_15_=2.96, *P*=.01) and reduced negative affect after (mean 11.69 [SD 3.34]) compared with that before watching (mean 13.63, [SD 5.12], *t*_15_=2.78, *P*=.014). Participants reported reduced positive affect after listening to happy music (mean 26.38 [SD 6.96]) compared with that before watching it (mean 29.56 [SD 5.17], *t*_15_=2.62, *P*=.02), but no significant difference for negative affect (*t*_15_=1.60, *P*=.13). There was no significant difference for neutral music for both affects, both *P* values were >.76.

#### Condition 3: Listen to Music While Walking

Participants reported an increased negative affect while walking and listening to happy music (mean 13.31 [SD 4.88]) compared with neutral (mean 15.00 [SD 5.44]) music (Z=2.64, *P*=.08). No other significant differences were observed, all *P* values were >.13.

#### Heart Rate

We planned to verify data obtained from PANAS and determine whether our participants experienced accelerated or decelerated heart rate as a result of emotional stimuli [15]. From the 50 participants, we had some data loss due to technical faults (n=9; 3 from Condition 1, 3 from Condition 2, and 3 from Condition 3), leaving us with data obtained from 41 participants. We first reviewed descriptive statistics and found that data was normally distributed. A one-way between-subjects analysis of variance was conducted to compare the effect of emotion (happy, sad, and neutral) on participants’ heart rates. We did not find any significant effect of emotion on their heart rate for the 3 conditions (*F*_2,120_=0.13, *P*=.88; see Table 3 for means and SD).

### Emotion Recognition

#### Happy Versus Sad

**Figure 2:**
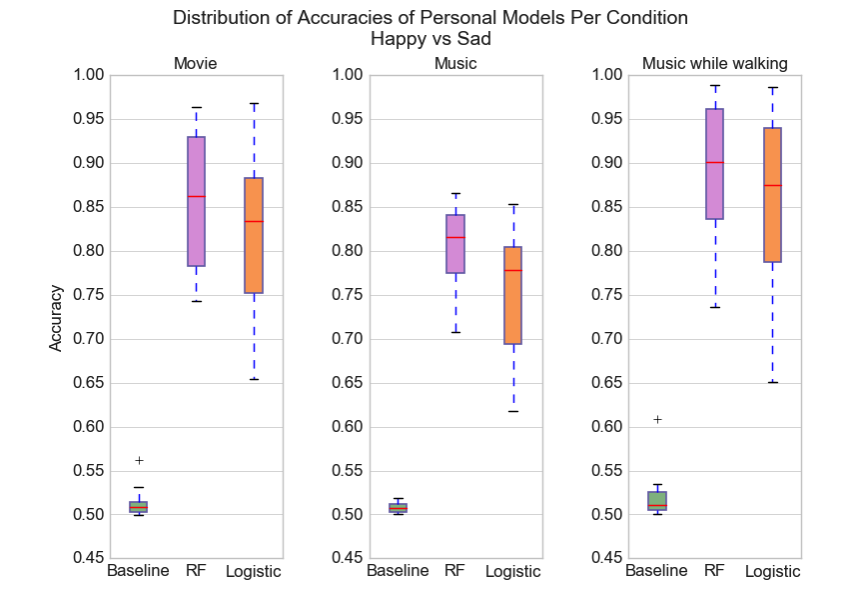
Boxplot of classification accuracies for participants divided by conditions. Algorithms tested were baseline (pick majority), random forests, and logistic regression. Outliers are indicated by +. The highest classification accuracies were achieved with Condition 1 (movie) and Condition 3 (music while walking). For all conditions, the models achieved accuracies greater than 78% for over half the users. RF: random forest.

Figure 2 illustrates 3 boxplot sets, one for each condition, showing distribution of accuracies for the personal model of each participant. For all 3 conditions, personal baselines have accuracies in the range 50%-54%. For all conditions, both random forest model and logistic regression outperformed the baseline with accuracies in the range 62%-99%. Condition 1 (movie) and Condition 3 (music while walking) resulted in the highest classification accuracies with median accuracies over 82%.

**Table 3:** Mean heart rate and SD in brackets for all 3 emotions.

Emotions	Mean (SD)
Happy	104.43 (14.55)
Sad	91.68 (16.31)
Neutral	105.77 (14.50)

**Table 4:** Average user lift and average personal model accuracy per condition.

Features and model					AUC^a^ (SD)		F1 score (SD)		Accuracy (SD)		User lift		*P* value
**Accelerometer, gyroscope, heart rate**													
	**Condition 1: Watch movie, then walk**												
		Baseline		0.500 (0.000)		0.348 (0.017)		0.513 (0.015)					
		Logistic regression		0.876 (0.085)		0.817 (0.089)		0.818 (0.089)		0.305		<.001	
		Random forest		0.923 (0.059)		0.854 (0.073)		0.854 (0.073)		0.342		<.001	
	**Condition 2: Listen to music, then walk**												
		Baseline		0.500 (0.000)		0.342 (0.007)		0.508 (0.006)					
		Logistic regression		0.812 (0.081)		0.748 (0.071)		0.748 (0.071)		0.240		<.001	
		Random forest		0.887 (0.046)		0.806 (0.047)		0.806 (0.047)		0.298		<.001	
	**Condition 3: Listen to music while walking**												
		Baseline		0.500 (0.000)		0.356 (0.031)		0.520 (0.027)					
		Logistic regression		0.900 (0.096)		0.849 (0.107)		0.849 (0.107)		0.329		<.001	
		Random forest		0.948 (0.057)		0.890 (0.081)		0.891 (0.080)		0.371		<.001	
**Accelerometer, heart rate**													
	**Condition 1: Watch movie, then walk**												
		Baseline		0.500 (0.000)		0.348 (0.017)		0.513 (0.015)					
		Logistic regression		0.809 (0.105)		0.752 (0.099)		0.753 (0.099)		0.240		<.001	
		Random forest		0.891 (0.081)		0.821 (0.090)		0.822 (0.089)		0.309		<.001	
	**Condition 2: Listen to music, then walk**												
		Baseline		0.500 (0.000)		0.342 (0.007)		0.508 (0.006)					
		Logistic regression		0.729 (0.070)		0.674 (0.055)		0.675 (0.055)		0.167		<.001	
		Random forest		0.847 (0.046)		0.768 (0.045)		0.769 (0.045)		0.261		<.001	
	**Condition 3: Listen to music while walking**												
		Baseline		0.500 (0.000)		0.356 (0.031)		0.520 (0.027)					
		Logistic regression		0.876 (0.095)		0.821 (0.106)		0.821 (0.106)		0.301		<.001	
		Random forest		0.933 (0.067)		0.871 (0.088)		0.871 (0.088)		0.351		<.001	
**Accelerometer**													
	**Condition 1: Watch movie, then walk**												
		Baseline		0.500 (0.000)		0.348 (0.017)		0.513 (0.015)					
		Logistic regression		0.786 (0.097)		0.726 (0.089)		0.727 (0.089)		0.215		<.001	
		Random forest		0.847 (0.076)		0.773 (0.077)		0.774 (0.077)		0.261		<.001	
	**Condition 2: Listen to music, then walk**												
		Baseline		0.500 (0.000)		0.342 (0.007)		0.508 (0.006)					
		Logistic regression		0.708 (0.056)		0.657 (0.047)		0.658 (0.047)		0.150		<.001	
		Random forest		0.783 (0.051)		0.712 (0.042)		0.713 (0.042)		0.205		<.001	
	**Condition 3: Listen to music while walking**												
		Baseline		0.500 (0.000)		0.356 (0.031)		0.520 (0.027)					
		Logistic regression		0.848 (0.086)		0.789 (0.096)		0.790 (0.095)		0.269		<.001	
		Random forest		0.899 (0.066)		0.825 (0.080)		0.825 (0.079)		0.305		<.001	

^a^AUC: area under the curve.

**Figure 3:**
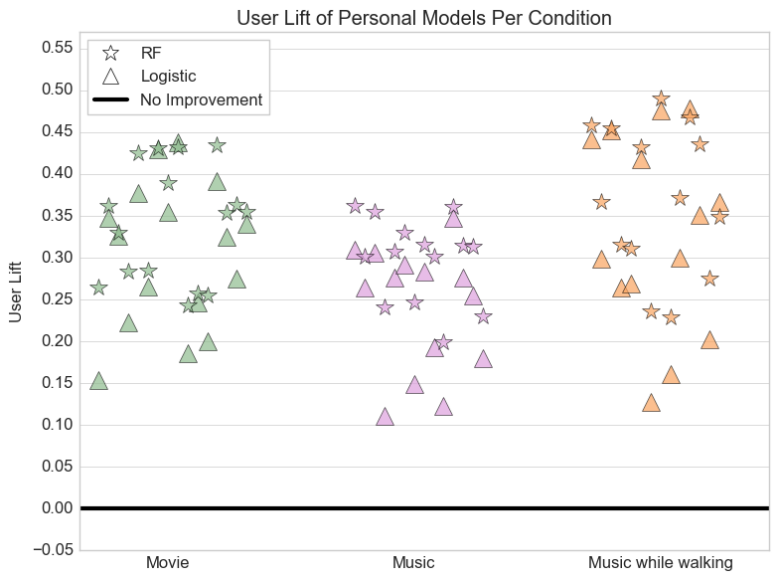
The user lift for personal models per condition. The random forest user lift is calculated as (random forest accuracy – baseline accuracy) and the logistic regression user lift is calculated as (logit accuracy – baseline accuracy). The personal models achieve higher accuracies than the personal baseline models.

We used the user lift framework to quantify whether a personal model was better than a personal baseline for each user [26]. We calculated the user lift as the difference in the accuracy of the personal classifier and the personal baseline (classifier accuracy–personal baseline accuracy). We used the nonparametric permutation test to determine whether user lifts had a mean greater than 0 (see Table 4). Figure 3 shows the calculated user lift for each participant using random forest model and logistic regression. We included this figure because average user lift can obscure the presence of negative user lift for some participants. Using features extracted from the accelerometer, gyroscope, and heart rate data resulted in the highest accuracies. Using only features from accelerometer data resulted in lower accuracies. Overall for the personal models, the average user lift was greater than 0 for all conditions, indicating that the trained personal models outperformed the baseline.

#### Happy Versus Neutral Versus Sad

**Figure 4:**
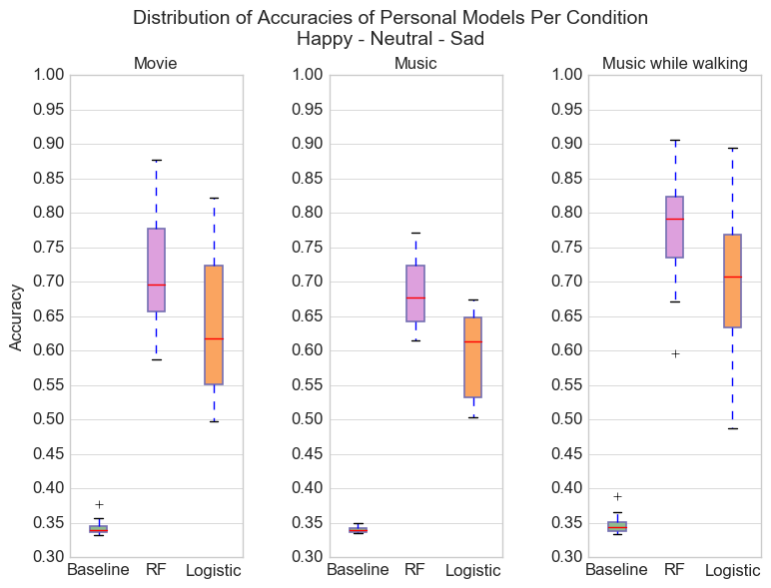
Classification accuracies for participants divided by conditions for the recognition of happiness, sadness, and neutral emotional states. The lower accuracies when recognizing the neutral emotional state indicates that the neutral walking data does have more similarities to the happy and sad walking data, which may indicate the need for additional features. RF: random forest.

Figure 4 shows the distribution of accuracies of personal models for the three-class classification task of predicting happy-neutral-sad emotional states. We used all features (acceleration, angular velocity, and heart rate) for classification. Although personal models on average outperformed the baseline, accuracies are lower than those achieved when predicting only happy versus sad. Because the number of samples for each class is approximately the same, the baseline predicting the majority class is able to classify correctly only about a third of testing samples. See Table 5 for user lift results. Personal models outperformed personal baselines, but overall accuracy was lower than binary classification of happy versus sad.

### Emotion Cross-Validation

**Figure 5:**
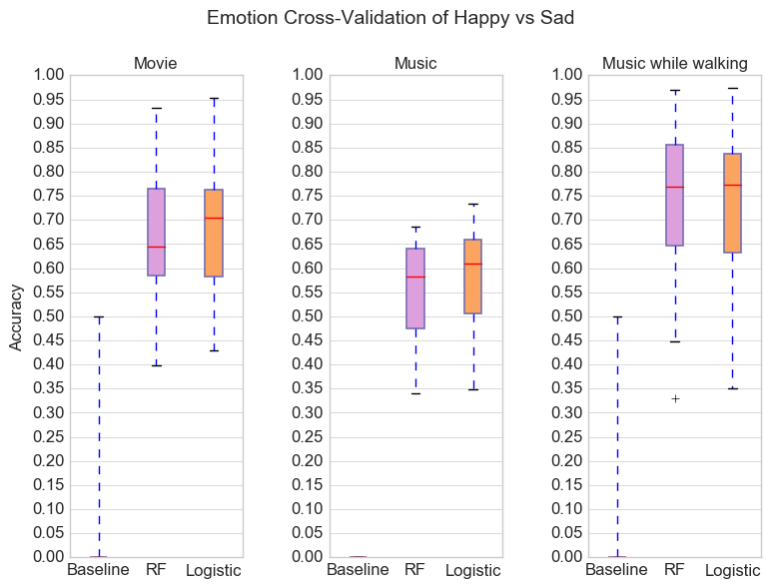
Boxplot of classification accuracies for participants divided by conditions. The results are for 10-fold cross-validation, with each fold in the training data consisting of contiguous windows from both happy and walking data, and the held-out test fold consisting of contiguous windows from either the happy or the sad walking data. RF: random forest.

We conducted an experiment to assess the effect of neighborhood bias in evaluation of our models using random cross-validation. In this experiment, we conducted 10-fold cross-validation for each personal model, but the testing fold that was held out during each iteration held out a contiguous happy data block or a contiguous sad data block. The goal was to determine with higher confidence whether classifiers were learning patterns associated with emotions, as opposed to just learning to distinguish between different walking periods. In addition, this type of validation takes into consideration neighborhood bias, which can lead to overly optimistic performance estimates [24]. Results (see Figure 5 and Table 6) show that accuracies across all conditions dropped compared with accuracies when using random cross-validation. Personal models outperformed personal baselines but overall accuracy was poor.

**Table 5:** Average user lift and average personal model accuracy per condition for the three-class classification task of predicting happy-neutral-sad.

Model		F1 score (SD)		Accuracy (SD)		User lift		*P* value
**Condition 1: Watch movie, then walk**								
	Baseline		0.175 (0.010)		0.343 (0.011)			
	Logistic regression		0.632 (0.103)		0.635 (0.103)		0.292	<.001
	Random forest		0.722 (0.090)		0.723 (0.090)		0.380	<.001
**Condition 2: Listen to music, then walk**								
	Baseline		0.173 (0.004)		0.340 (0.004)			
	Logistic regression		0.591 (0.062)		0.594 (0.061)		0.254	<.001
	Random forest		0.684 (0.048)		0.685 (0.047)		0.345	<.001
**Condition 3: Listen to music while walking**								
	Baseline		0.180 (0.014)		0.348 (0.015)			
	Logistic regression		0.709 (0.113)		0.711 (0.113)		0.363	<.001
	Random forest		0.781 (0.087)		0.782 (0.087)		0.434	<.001

**Table 6:** Average user lift and average personal model accuracy per condition.

Model		F1 score (SD)	Accuracy (SD)	User Lift	*P* value
**Condition 1: Watch movie, then walk**					
	Baseline	0.031 (0.121)	0.031 (0.121)		
	Logistic regression	0.787 (0.104)	0.682 (0.139)	0.650	<.001
	Random forests	0.763 (0.112)	0.651 (0.146)	0.620	<.001
**Condition 2: Listen to music, then walk**					
	Baseline	0.000 (0.000)	0.000 (0.000)		
	Logistic regression	0.705 (0.099)	0.575 (0.115)	0.575	<.001
	Random forests	0.678 (0.105)	0.543 (0.118)	0.543	<.001
**Condition 3: Listen to music while walking**					
	Baseline	0.036 (0.129)	0.036 (0.129)		
	Logistic regression	0.812 (0.140)	0.723 (0.179)	0.688	<.001
	Random forests	0.815 (0.148)	0.731 (0.185)	0.695	<.001

**Table 7:** Accuracy scores for leave-one-user-out cross-validation.

Model		AUC^a^ (SD)	F1 score (SD)	Accuracy
**Condition 1: Watch movie, then walk**				
	Baseline	0.500 (0.000)	0.342 (0.021)	0.508 (0.018)
	Logistic regression	0.539 (0.137)	0.461 (0.112)	0.515 (0.090)
**Condition 2: Listen to music, then walk**				
	Baseline	0.500 (0.000)	0.332 (0.011)	0.499 (0.010)
	Logistic regression	0.539 (0.084)	0.467 (0.061)	0.519 (0.059)
**Condition 3: Listen to music while walking**				
	Baseline	0.500 (0.000)	0.323 (0.034)	0.490 (0.032)
	Logistic regression	0.510 (0.173)	0.476 (0.092)	0.505 (0.082)

^a^AUC: area under the curve.

However, the performance of models remains higher than personal baselines with the exception of a few users. Only a quarter of baseline models under Condition 1 and Condition 3 achieved accuracies ranging from 0 to 0.5; the rest have accuracies of 0. This is expected because a baseline model predicted on the majority class will achieve an accuracy of 0 when tested on a contiguous block of the opposite class.

We conclude that for at least half the participants in Condition 1 (movie) and Condition 3 (music while walking), models are likely learning patterns associated with sad and happy emotions. In addition, high accuracies indicate that model performance is not a result of neighborhood bias [24].

### Generalizing Across Users

We conducted leave-one-user-out cross-validation to assess how well a model trained on data from certain users would be able to generalize to a user for whom no data are available. We compared both the logistic regression and random forest model. However, random forest models performed similarly or worse than logistic regression; therefore, we only discussed results of the best performing logistic regression compared against the baseline (see Table 7). Logistic regression performed poorly across all conditions, showing that using data from different users to do emotion recognition on a different user is not possible with current features and logistic regression. Low accuracies across all conditions show that the behavior from user to user varies considerably, even when performing a similar action. Owing to the small number of users per condition (<18), data may not be enough to make accurate predictions for users not included in the training set [24]. However, it also highlights a limitation in our modeling approach, in that different features or more advanced models may be necessary to generalize across users. Ideally, deployment of an app should include an initial data collection and calibration phase, which can be used to build a high accuracy personal model for each user.

### Model Interpretability

We address model interpretability, that is, how models are able to differentiate between emotions, by examining information gain of features. Random forest models can be interpreted by examining feature importances, and logistic regression can be interpreted by the sign and value of the coefficients. Random forest models outperformed logistic regression in our results; therefore, we limit our analysis to feature importances of random forest models.

**Figure 6:**
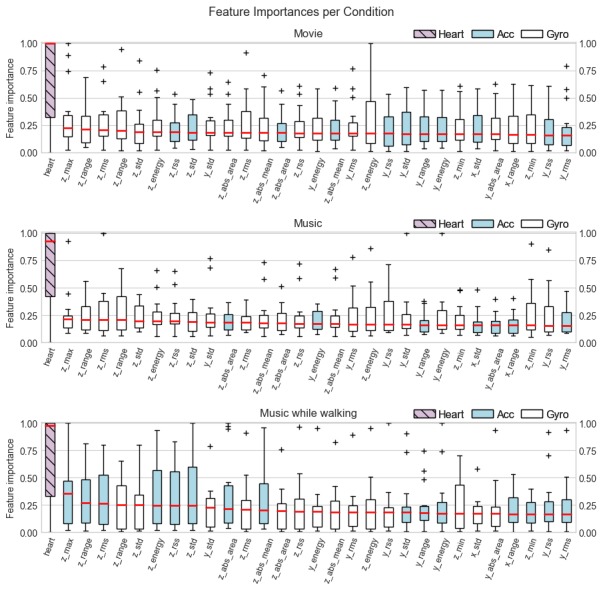
Distribution of feature importances per feature for all personal models. Acc: accelerometer; Gyro: gyroscope.

Because we are building personal models, features that might be important for one user may be less important for another user. To show this, we plotted the distribution of feature importance values for each feature across all users using boxplots, as seen in Figure 6. Boxplots are sorted by median and we included only the top 30 features for visibility with the trend of the remaining features being about the same. To obtain feature importances for each user, we computed the mean feature importance for each feature in cross-validation folds and divided each feature by the maximum feature importance value. Thus, a value of 1.0 indicates that a feature was the most important among all the features.

A compact boxplot indicates that the feature has similar importance across all users. On the other hand, a boxplot with a large spread indicates that the feature is important for some users but less important for other users. For all conditions, heart rate was the most important feature. In fact, for Condition 1 (movie), heart rate was the most important feature for at least half of users (median=1.0). The rest of the features have distributions with smoothly decreasing medians but with heart rate being the only feature with a clear difference from other features.

## Discussion

### Behavioral Response to Stimuli

Participants reported feeling less negative affect after watching sad videos or after listening to sad music. This is contrary to the other condition (listening to music while walking) in which participants reported feeling more negative during happy stimuli compared with the neutral ones. Our findings suggest that the walking activity after experiencing a stimulus is useful to alleviate negative mood, similar to that reported [27,28]*,* but not while experiencing stimuli. One reason for this is that participants were focused on the song, and possibly, the change between music types creates resentment or unhappiness. Some studies suggest that people may prefer sad music [29,30], which may influence participants’ response toward stimuli. However, a subset of 10 participants reported liking the sad stimulus the least compared with happy and neutral stimuli, suggesting that this is not a case of liking sad music more than others. This personal preference self-report further adds credence to PANAS results that walking is useful in alleviating negative mood.

From heart rate data, our participants did not experience any significant difference in heart rate between emotions. One possible explanation is that walking itself is a vigorous activity compared with standing still; thus, the brief exposure to emotional stimulus may not have been captured holistically. The other possible explanation is that both emotions were equally successful in evoking their emotional states; therefore, there was a nonsignificant difference between them. Nonetheless, data from PANAS suggest that it is likely the latter because participants reported experiencing a difference between positive and negative states.

### Classifiers for Emotion Recognition

High accuracies achieved across all users for classification of happy versus sad emotional states provide further evidence for the hypothesis that movement sensor data can be used for emotion recognition. To build personal models, we used statistical features that are computationally cheap, which would make it feasible to deploy a smart watch or a mobile phone app that can track emotions from movement sensor data without taxing the smart watch or mobile phone processor.

Using only accelerometer data for emotion recognition resulted in mean AUC values of at least 71% for all conditions. The combination of accelerometer data features and heart increased the overall performance of models to a mean AUC of 73%. The use of accelerometer, heart rate, and gyroscope features increased the mean AUC to 81%. This provides a strong motivation to use gyroscope and heart rate data in applications attempting to infer emotional states from movement data, especially given that application programming interfaces of mobile phones and smart watches make it easy to retrieve gyroscope and heart rate. In addition, the high importance of the heart rate feature in random forest models ought to encourage developers to use heart rate data from a smart watch for emotion recognition.

When comparing the classification results using features extracted from all sensor data on classification of happy versus sad emotions, we achieved high-fidelity emotion recognition models with an accuracy of ≥80% for 62.5% (55/88) of the personal models, average-fidelity models with an accuracy between 70% and 80% for 27.3% (24/88) of the personal models, and low-fidelity models with an accuracy of <70% for 10.2% (9/88) of the personal models. These results are encouraging. However, they also indicate that further work is needed to achieve consistent results across different users and accuracies closer to 100%. For example, this could be achieved by extracting additional features, using a more complex classifier, or by collecting more data for training and testing personal models. Lastly, our results on emotion cross-validation highlight that personal models for about half the participants are learning features that capture emotions.

### Limitations

Previous studies have utilized a contrast experimental paradigm to manipulate the following participants’ moods: positive versus negative mood [2]; negative or neutral [31]; positive, negative, and neutral [32,33] using music or avatars. Past research findings indicate that negative moods tend to reduce mood recovery and a slower response for accurately identifying other emotional expressions [20,31]. Although these user studies did not apply to emotion recognition from sensor data obtained from a smart watch, we did not address issues, such as reduced mood recovery, for participants who were shown the sad stimulus first; however, we did perform counterbalancing for our stimuli on our participants.

The integrity of sensor data is a concern. For Conditions 1 and 2, participants were primed with audio and audiovisual stimuli for a few minutes, but beyond PANAS scores, we do not have other means to indicate that the stimulus had the intended effect. Furthermore, the effect of the stimulus on participants is questionable given that participants were not emotionally invested in movie and music clips that were shown. Personal models do distinguish at high accuracies between features extracted from happy, sad, and neutral emotions, but we do not know for certain that happy data is truly associated with a “happy” emotional state in users. In general, given that the mixed-design study consisted of 3 conditions, 50 participants is a small sample size.

From a modeling and data analysis point of view, the amount of data collected was small. Hence, this limits the training and validation of classifiers. Although personal models yielded high accuracies for many users, for other users, the results were slightly better than random guessing. Finally, we did not consider more flexible modeling approaches, such as using a time-aware model or using a neural network trained on raw sensor data, instead of extracting features from sliding windows.

The personal models we built are naïve, in that each window is an independent sample. Therefore, a model could potentially predict happy-sad-happy for 3 consecutive one-second windows, which is unrealistic as a user is not likely to go from happy to sad and back to happy in a matter of 3 seconds. This limitation of our modeling approach will be addressed in future work.

### Comparison With Prior Work

Our work is closest to the work reported previously [10,11]. In [10], the details of the design study are omitted, including the choice of videos and procedures. A limitation in a previous study [10] is that data was collected from two mobile phones, one strapped to the wrist and one strapped to the ankle of participants. In a previous study [11], 123 participants were recruited (twice the size of our sample), and smart bracelets were used for data collection with participants wearing a smart bracelet on the wrist and another smart bracelet on the ankle (with the latter violating ecological validity). We achieved accuracies comparable to those reported in another study [11], using only data from one smart watch on participants’ wrists and without relying on data from other body locations. Our work also differs in that we focus on happy and sad emotional states, whereas in a study [11], researchers focused on happy and angry emotional states. In contrast to prior work, we performed more rigorous testing by including emotion cross-validation and by extracting features from an accelerometer, a gyroscope, and heart rate sensors.

In contrast to emotion prediction based on typing behavior [5,6], mobile phone usage [7,8], and mobile phone speech recordings [9], we focused on movement data and heart rate data. The EmotionSense system does use accelerometer data to determine whether a user is moving but not for emotion recognition [9].

### Conclusions and Future Work

Our findings suggest that emotional expression is transparent even in automatic functions such as walking gait. This finding is interesting in that healthy young adults typically do not report large differences in their emotional state, unlike some clinical groups [34]. These findings also validate our methodological approach with respect to priming the emotional state and the subsequent modeling using machine-learning algorithms.

Many studies have focused on face and voice modalities, but recent studies have shown that we tend to adopt different body postures and gaits as a reflection of our emotions and that these postures and gaits are just as easily recognized by others, indicating that walking gait is a form of social signal. However, the emotional behavioral response is only evident after experiencing the stimulus on its own or while experiencing both together (eg, listening to music while walking). Nonetheless, our findings provide further knowledge in the field of social communication, particularly in specific clinical conditions. The unobtrusive wearable is a good complement for collecting data and for providing biofeedback and interventions for emotional regulation. Recent studies have started analyzing the possibility of using wearables to provide more readily available treatment for patients and provide feedback to clinicians to cater to their needs [34-36]. Benefits of using these wearables, particularly in identifying emotional states, are useful for diagnosis or monitoring specific clinical conditions, such as social anxiety and borderline personality disorder. Although most research is focused on getting patients to self-rate their moods, having actigraph data and walking patterns will complement the information necessary for clinicians. Other than for a clinical population, this type of information is also useful for vulnerable populations (eg, older adults) experiencing some emotional distress and social isolation [37]. Future studies should look into the duration of having on such wearables (over 24-hour cycles) and duration in experiencing stimuli (acute or chronic experiences).

## Appendix

Multimedia Appendix 1 Means and SDs in brackets for positive and negative affect scores for each emotion.

## Abbreviations

AUC: area under the curve
