# Supplementary material for: Emotion Recognition Using Smart Watch Sensor Data: Mixed-Design Study
Source: JMIR Ment Health. 2018 Aug 8;5(3):e10153. doi: 10.2196/10153 (PMC6105867; doi:10.2196/10153)
Supplement: Multimedia Appendix 1 [file mental_v5i3e10153_app1.pdf]

Multimedia Appendix 1

Means and standard deviations in brackets for positive and negative affect scores for each emotion.

	Happy				Sad				Neutral			
	Positive		Negative		Positive		Negative		Positive		Negative	
	Before	After	Before	After	Before	After	Before	After	Before	After	Before	After
Condition 1: Watch movie then walk	27.59 (6.06)	26.65 (7.68)	16.65 (7.45)	14.65 (5.99)	24.88 (6.71)	24.76 (8.46)	19.00 (7.20)	14.94 (6.79)	24.24 (6.98)	23.24 (7.48)	15.35 (6.96)	14.41 (6.40)
Condition 2: Listen to music then walk	29.56 (5.18)	26.38 (6.96)	11.75 (3.34)	11.31 (3.05)	20.31 (5.79)	24.00 (5.33)	13.63 (5.12)	11.69 (3.34)	23.69 (7.05)	24.13 (6.82)	12.19 (3.69)	12.13 (3.30)
Condition 3: Listen to music while walking		32.38 (8.33)		13.31 (4.88)		29.31 (8.40)		14.81 (5.27)		30.88 (7.26)		15.00 (5.44)
